# Micron-Size White Bamboo Fibril-Based Silane Cellulose Aerogel: Fabrication and Oil Absorbent Characteristics

**DOI:** 10.3390/ma12091407

**Published:** 2019-04-30

**Authors:** Dinh Duc Nguyen, Cuong Manh Vu, Huong Thi Vu, Hyoung Jin Choi

**Affiliations:** 1 Department for Management of Science and Technology Development, Ton Duc Thang University, Ho Chi Minh City 700000, Vietnam; nguyendinhduc@tdtu.edu.vn; 2 Faculty of Environment and Labour Safety, Ton Duc Thang University, Ho Chi Minh City 700000, Vietnam; 3 Center for Advanced Chemistry, Institute of Research and Development, Duy Tan University, Da Nang 550000, Vietnam; 4 Faculty of Chemical-Physical Engineering, Le Qui Don Technical University, 236 Hoang Quoc Viet, Ha Noi 100000, Vietnam; 5 AQP research and control pharmaceuticals Joint Stock Company (AQP Pharma J.S.C) Dong Da, Ha Noi 100000, Vietnam; huongvtaqp@gmail.com; 6 Department of Polymer Science and Engineering, Inha University, Incheon 22212, Korea

**Keywords:** cellulose aerogel, oil absorbent, cellulose, white bamboo fibril, water pollution

## Abstract

Micron-size white bamboo fibrils were fabricated from white bamboo and used as a source for the production of highly porous and very lightweight cellulose aerogels for use as a potential oil absorbent. The aerogels were fabricated through gelation from an aqueous alkali hydroxide/urea solution, followed by a conventional freeze-drying process. The morphology and physical properties of the aerogels were characterized by field emission scanning electron microscopy and Brunauer–Emmett–Teller surface area analysis, respectively. Successful silanization of the cellulose aerogel was confirmed by energy-dispersive X-ray spectroscopy, Fourier-transform infrared spectroscopy, and water contact angle measurements. The fabricated silane cellulose aerogel exhibited excellent absorption performance for various oil and organic solvents with typical weight gains ranging from 400% to 1200% of their own dry weight, making them promising versatile absorbents for a range of applications, including water purification.

## 1. Introduction

Water pollution caused by oil spillage related to the rapid development of the petroleum industries have serious effects on the environment and human life [1,2,3]. To resolve this problem, many methods have been proposed for water purification, such as water/oil separation [4,5,6], photocatalytic degradation [7,8], and adsorption [9,10,11]. Among these technologies, the use of porous absorbents with a hydrophobic surface is very promising for the rapid removal of oil from the surface of water. Various types of materials used as absorbents for cleaning oil spills have been fabricated. Oil absorbents can be sorted as inorganic minerals, synthetic organics, and natural organic materials [12]. Inorganic materials, such as fly ash and exfoliated graphite have low oil absorption capacity, whereas synthetic organic materials (i.e., polypropylene and polyurethane) possess high affinity to oil and organic solvents but cause a waste problem after their use due to their very slow degradation. Natural organic materials for oil absorption from plants (cellulose fibers) and animal residues (chitin and chitosan) have attracted increasing attention because of their renewability, low-cost, and biodegradability [13]. In addition, many kinds of natural materials, such as kapok fiber [14,15], cotton fiber [16], wool fiber [17], milkweed [18], and sawdust [19], have been exploited for the simple, effective, and inexpensive treatments of oil spills. However, most of these are hydrophilic, resulting in low oil sorption capacity. Therefore, there is still a need to find new environmental friendly absorbents with high oil absorption capacity, good selectivity, and low-cost. 

Aerogels are a highly porous solid that hold up to 99% by volume of air within their pores and are known as the lowest density solid material [20]. These materials have become the most promising absorbents owing to their lightness, high porosity, and large inner surface area. Aerogels can be obtained from both inorganic sources, such as silica [21,22,23] and metal oxides [24], and organic sources [25,26,27]. Generally, the mechanism of oil sorption by aerogels is considered to be governed mainly by aerogel surface adsorption, absorption, and diffusion through the voids via interfiber capillary action [28,29,30], and the amount adsorbed is known to be dependent on surface area and porosity of adsorbents [31].

Concurrently, as an abundant, sustainable, renewable, and biodegradable resource of biopolymer, various cellulose-based aerogels have been investigated [32,33,34,35,36,37]. However, because the untreated cellulose aerogels have high hydrophilicity, they can absorb both oil and water during the absorption processing [36]. Therefore, the surface modification is needed to improve the hydrophobicity and absorption efficiency. The silanization processing also became one of the best ways for this aim [35,36]. Cellulose can be obtained from plants, such as bamboo. In Vietnam, bamboo is distributed widely in the north and is mainly being used as a raw material for tradition products such as toothsticks, chopsticks, floors, and some furniture. To the best of the authors’ knowledge, there are few reports of the fabrication of aerogels from white bamboo and their application as both an absorbent for oil and other toxic chemicals especially at the time when water pollution by oil and chemical spills is becoming a serious problem caused by the huge development of the petrochemical industry. 

In this study, bamboo was used as a source of micron-size white bamboo fibrils prior to fabricating the aerogel. The aerogels were prepared in a simple way from the gels of micron-size white bamboo fibrils (MWBFs) in an aqueous alkali hydroxide/urea solution, followed by conventional freeze-drying. The resulting aerogel was rendered hydrophobic and oleophilic after being treated with a silane compound using a common chemical vapor deposition process. 

## 2. Experiments

### 2.1. Materials and Chemicals

Dendrocalamus membranaceus Munro (white bamboo, ~3 years) from Hoa Binh Province, Vietnam was used as a micron-size fiber resource. Gama-methacryloxypropyltrimethoxysilane (MEMO silane) was purchased from Evonik Industries (Ho Chi Minh, Vietnam). All other chemicals, including alkali hydroxide, urea, and ethanol, were of reagent grade (Xilong Chemical Co. Ltd, Guangdong, China). All aqueous solutions were prepared using distilled water.

### 2.2. Preparation of Micron-Size White Bamboo Fibrils

**Figure 1:**
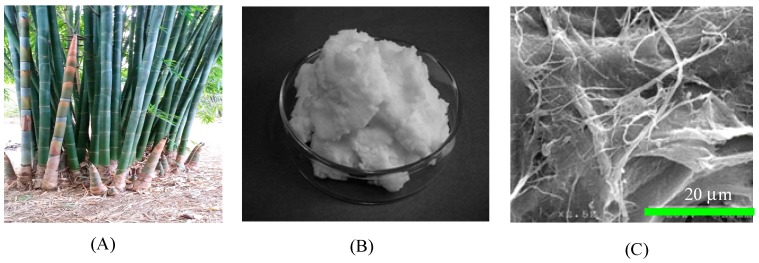
Picture of white bamboo (**A**), micron-size white bamboo fibrils (MWBFs) (**B**), and SEM image of MWBFs (**C**).

The MWBF was fabricated from raw white bamboo using both a steam explosion following alkaline treatment technique and the mechanical extraction method (microgrinding). Raw white bamboo (~3 years old) was first cut into bamboo culms of 50–60 cm in length using a saw machine, and placed into an autoclave with over-heated steam at 175 °C and 0.7–0.8 MPa for 60 min. The steam was then released suddenly for 5 min and the cycles of sudden steam release were repeated 9 times. Subsequently, samples were immersed in a 2% NaOH solution at 70 °C for 5 h to ensure the complete removal of the cell walls. The roller looser was then used to extract the slabs into small fibers. Finally, they were washed with fresh water until they were neutralized, and dried in an oven for 24 h at 105 °C. The resulting fibers were dispersed in water with a fiber content of 10 wt.%. They were then cut into pulp fibers using a food mixer. The pulp fibers were passed 15 times between static grind and rotating grind stones revolving at 1500 rpm (MKCA6-3, Masuko Sangyo Co. Ltd., Saitama, Japan). The obtained MWBF (water slurry with 90% water) was treated with ethanol to remove the water and filtered using a vacuum pump to obtain a sheet of MWBF. The filtered sheet of MWBF was stirred with an additional amount of ethanol using a stirrer at 5000 rpm for 15 min. The morphology of the MWBF was examined by scanning electron microscopy (SEM) (JEOL, Tokyo, Japan), as shown in Figure 1. The SEM in Figure 1 indicates that the diameter of cellulose fiber is in range from 90 nm to 0.2 µm, but its length is in the order of tens of micron. 

### 2.3. Preparation of Cellulose Aerogel

The solvent mixture of NaOH/urea/H_2_O (7:12:81 w/w) was precooled to approximately 5 °C. The desired amount of MWBF samples (1.5, 2, and 2.5 wt.%) was then dispersed immediately into the solvent system under vigorous stirring at this low temperature until a semitransparent or transparent gel was achieved, depending on the MWBF concentration. At the final stage, the gel was vacuumed to remove the air bubbles. The specimen thickness was controlled to approximately 2 cm using a beaker as a mold and then immersed into ethanol to obtain the 5 wt.% solution. A 100 mL of aqueous 10 wt.% H_2_SO_4_ solution was then added at ambient temperature for coagulation. The resulting cellulose hydrogels were washed with excess distilled water to remove the residual chemical reagents. The sample was then frozen in a freezer at −80 °C for 24 h and freeze-dried using a FTS Systems Dura-Stop Digital Control Stoppering Tray Dryer to obtain the desired cellulose aerogel (CA).

### 2.4. Fabrication of Silane Treated Cellulose Aerogel 

A thermal chemical vapor deposition technique was used for surface modification of the cellulose aerogel. A Petri dish containing MEMO silane was placed in a vacuum desiccator together with the aerogel samples. The desiccator was sealed and vacuumed to 0.01 MPa, and then heated in an oven at 110 °C for various periods of time to determine the optimal conditions for the silanization reaction. Subsequently, the silane-coated cellulose aerogel (SCA) sample was kept in a vacuum oven for 30 min to remove the excess unreacted silane and by-products. 

### 2.5. Characterization

All the tests were carried out in triplicate and the average results are reported. Initially, the densities of the cellulose aerogels were calculated by measuring the mass and volume of the aerogels. The mass was measured using an analytical balance, Fisher Scientific Accu-225D, with accuracy of 0.1 mg. The volume was determined by measuring the dimensions using a digital Vernier caliper. Average density was estimated after 5 measurements for 3 different aerogels [26]. The porosity was calculated using Equation (1):

(1) $$\mathrm{Porosity}\;\left(\%\right)=\;\left(1-\frac{\rho_{a}}{\rho_{c}}\right)\times 100$$

where $\rho_{a}$ is the density of the aerogel and $\rho_{c}$ is the density of MWBF (1.59 g/cm^3^).

The BET specific surface area was determined by a N_2_ physisorption method using Gemini VII 2390 equipment (Micromeritic Instrument Co., Norcross, GA, USA). The wettability of the SCA was evaluated by measuring the water contact angle. Images of distilled droplets on the SCA surface were taken with a digital camera (Cannon 20D and Nikkon 105 mm 1:2.5 lens, Bangkok, Thailand) and imported into the measurement software. The volume of the droplet was fixed using a 5-mL cylinder. The software is licensed image processing and analysis in Java (ImageJ) [38] and included low-bond axisymmetric drop shape analysis (LB-ADSA). The mean of the three measurements performed at different surface locations are reported as the water contact angle. The Fourier-transform infrared (FTIR) spectra of the cellulose aerogel (CA) sample before and after silanization were recorded on IRAFFINITY-1S equipment (Shimadzu, Kyoto, Japan) at room temperature. The microstructure and elemental analysis of the uncoated and coated CA were examined by field-emission scanning electron microscopy (FE-SEM) (JEOL JSM-7600F, Tokyo, Japan) equipped with an energy-dispersive X-ray spectroscope. The sample was coated with a thin layer of platinum by sputtering.

To investigate its compressive properties, a cylinder sample with a diameter of 20 mm and a height of 13 mm was compressed to 80% of its original height by using a universal testing machine (Instron, 100 kN, Norwood, MA, USA) with a compressing speed of 10 mm. min^−1^. Five samples were tested to calculate the average value.

### 2.6. Oil/Solvent Absorption Capacity Measurements

The absorption capacity of SCA for various oils and organic solvents was determined by dipping a piece of SCA directly into the liquid (oil or solvent) for a certain time. The wet sample was then removed from the liquid and weighed after the aerogel surface has been blotted with filter paper to remove the excess surface oil/solvent. The test was repeated several times until the absorption process reached equilibrium. The absorption capacity (Q) was calculated from the mass gain using

(2) $$Q\;\left(\%\right)=\frac{W-W_{0}}{W_{0}}\times 100$$

where *W*_0_ and *W* are the weights of the SCA before and after absorption, respectively.

The pseudo-first-order model (Equation (3)) and pseudo-second-order model (Equation (4)) were used to evaluate the absorption kinetics, where *k*_1_ (h^−1^) and *k*_2_ (g·g^−1^·%^−1^·h^−1^) are the adsorption rate constants of the pseudo-first-order equation and the pseudo-second-order equation, respectively. In addition, both *Q_m_* and *Q_t_* are absorption capacities at equilibrium conditions and at time *t*, respectively.

(3) $$\ln\frac{Q_{m}}{Q_{m}-Q_{t}}=k_{1}t$$

(4) $$\frac{t}{Q_{t}}=\frac{1}{Q_{m}}t+\frac{1}{k_{2}Q_{m}^{2}}$$

To examine their reusability, the oil/organic swollen samples were squeezed by hand to remove the absorbed solvent. The weights of the aerogels before organic adsorption, after adsorption, and after squeeze for removal of organic were measured during each cycle. Five samples were tested for each experiment.

## 3. Results and Discussion

### 3.1. Cellulose Aerogel Characteristics

By altering the concentration of the MWBF dispersion from 1.5 to 2.5 wt.%, aerogels with different porosities were prepared using a freeze-drying method. Under freeze-drying conditions, a slight shrinkage was observed in these aerogels compared to their initial hydrogel dimensions. The experiments showed that the dispersion with 2.5 wt.% MWBF had very high viscosity, making it difficult to remove the air bubbles, resulting in a poor physical property. Table 1 lists the physical properties of the obtained cellulose aerogel.

**Figure 2:**
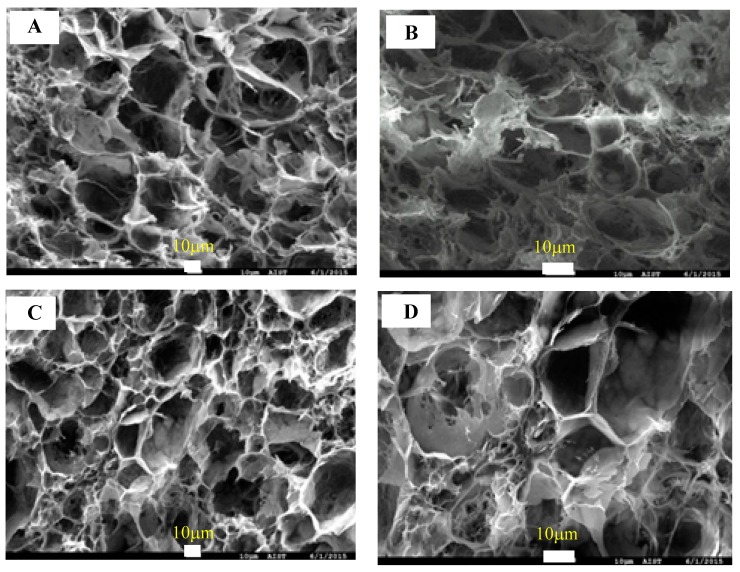
Field-emission scanning electron microscopy (FE-SEM) images of cellulose aerogel 2 wt.% (**a**,**b**) and cellulose aerogel 1.5 wt.% (**c**,**d**).

As shown in Table 1, the densities of the cellulose aerogels ranged from 0.085 to 0.144 g/cm^3^, and all cellulose aerogels exhibited very high porosity (>90%). On the other hand, the specific surface area exhibited the low values from 8.155 to 13.419 m^2^/g. Actually, this is quite common for aerogels obtained through a freeze-drying process. The main point here is that the typical porosity of such system is on the macron-scale, and hence the resulting specific surface area value is modest. The similar trend was also reported by Wang et al. [39]. Figure 2 shows the microstructure of a cross-section of the cellulose aerogel at different magnifications. The cellulose aerogels possessed a highly open porous honeycomb-like structure with a pore size distribution varying over a wide range from several to tens of micrometers. In addition, a network of interconnected uniform cellulose fibers appeared on the surface of the pore wall. 

### 3.2. Mechanical Properties of Aerogel

**Figure 3:**
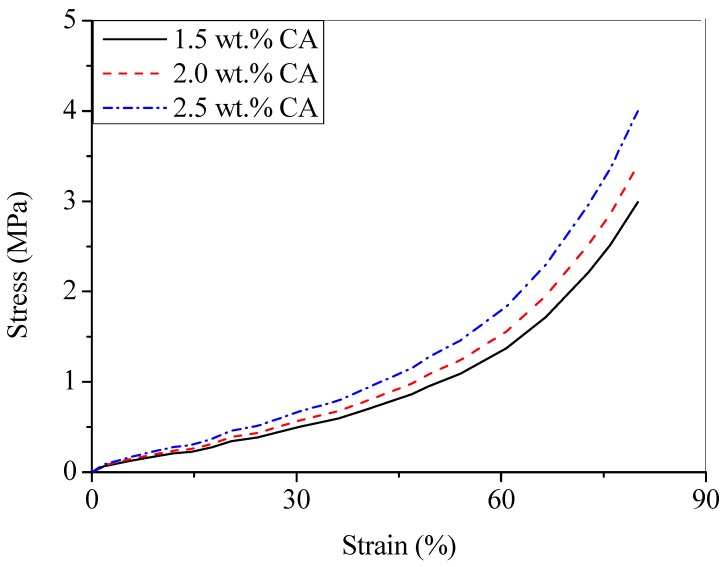
Typical compression stress–strain curves of cellulose aerogel (CA) with various cellulose concentration.

The typical compression stress–strain curve of CA with different cellulose content is shown in Figure 3.

Figure 3 could be explained by two stress regions with increasing cellulose content. The first region appeared before 60% strain and can be characterized by slowly increasing stress, while the second region appeared at above 60% strain with rapidly increasing stress. The stress of CA obtained from 1.5, 2.0, and 2.5 wt.% of cellulose concentration at 60% strain was 1.37 ± 0.01; 1.55 ± 0.01, and 1.83 ± 0.02 MPa, respectively. At 80% strain, the stress of corresponding cellulose aerogels increased to 2.99 ± 0.02, 3.41 ± 0.01, and 4.01 ± 0.02 MPa, respectively. These results mean that the stress increased with increasing cellulose concentration as a result of higher crosslinking density. In addition, the slope of stress–strain curves was also increased with increasing cellulose content in the CA. The slope of stress–strain corresponded to the compressive modulus and the internal structure of aerogel. The high crosslinking density also was considered as a reason of increment of the compressive modulus above.

### 3.3. Silane Modification of Cellulose Aerogel

As hydrophilicity is the inherent nature of cellulose due to its hydroxyl groups, a silane coating was carried out for cellulose aerogels to make them both hydrophobic and oleophilic. To achieve this, a simple thermal chemical vapor deposition procedure was performed for the aerogel with MEMO silane at 110 °C in a vacuum desiccator, as described in the above. To become “active”, the silane must first be hydrolyzed. The reaction of the silicon end of the molecule was initiated by the hydrolysis of the alkoxy group, usually after exposure to ambient moisture to form a silanol that releases alcohol as follows:

$$-\mathrm{Si}{\left({\mathrm{OCH}}_{3}\right)}_{3}+H_{2}O\to -\mathrm{Si}{\left(\mathrm{OH}\right)}_{3}+3{\;\mathrm{CH}}_{3}\mathrm{OH}$$

**Figure 4:**
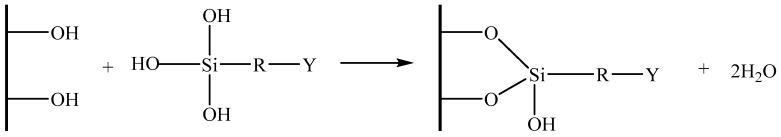
Silanization processing.

Once in the silanol state, the silane can be condensed on the aerogel surface, forming a direct covalent bond with the surface. The silanization process of the interior surface of as-prepared cellulose aerogel and silanized cellulose aerogel was shown in Figure 4.

**Figure 5:**
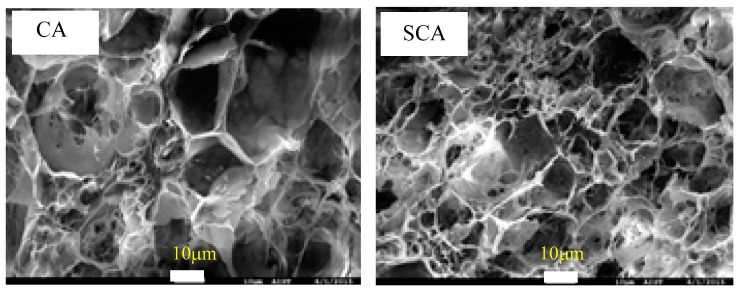
FE-SEM images of cross-section of CA and silane-coated cellulose aerogel (SCA).

As shown in Figure 5, the open porous microstructure of the cellulose aerogel was preserved after coating. In addition, there was no change in the surface of the cellulose aerogel after coating, which might be due to the very thin silane coating layer.

**Figure 6:**
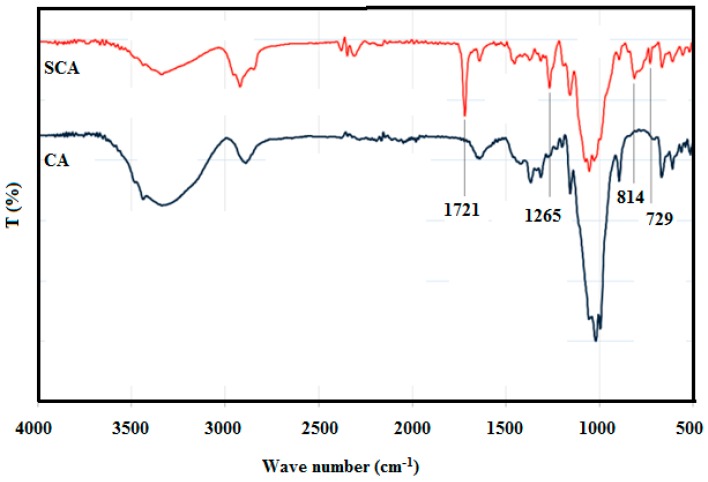
FTIR spectrums of cellulose aerogel and cellulose aerogel (CA) silanization with MEMO silane (SCA).

Successful silanization on the surface of the cellulose aerogel was confirmed by FTIR spectroscopy, as shown in Figure 6. The FTIR spectrum of SCA showed four new peaks compared to the spectrum of the uncoated aerogel. The absorption bands at ~731 cm^−1^ and ~1269 cm^−1^ were attributed to the stretching and bending vibrations of the C–Si linkage, respectively. This confirmed the condensation of silane on the CA surface. In addition, the peak at 1720 cm^−1^ on the SCA spectrum was assigned to the characteristic vibrations of the carbonyl group of MEMO silane attached to the aerogel surface. The absorption band at ~815 cm^−1^ was attributed to the vibration of the Si-O-Si linkage, which might have formed due to the self-condensation of the silanols.

**Figure 7:**
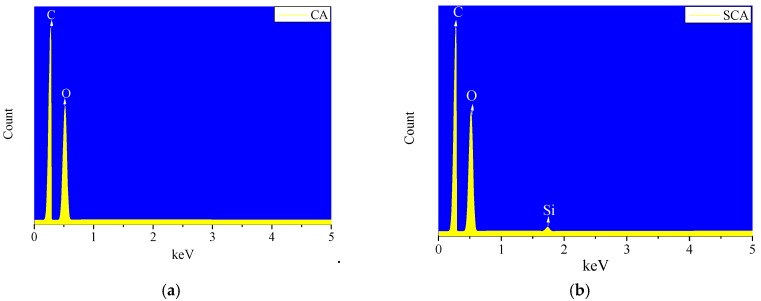
Energy dispersive X-ray spectroscopy (EDX) spectrum of (**a**) cellulose aerogel (CA) and (**b**) cellulose aerogel silanization with MEMO silane (SCA).

Silanization was confirmed by energy dispersive X-ray spectroscopy (EDX). The EDX spectrum of the uncoated CA revealed carbon and oxygen peaks but no silicon peak. After silanization, EDX showed peaks for carbon, oxygen, and silicon, as shown in Figure 7.

### 3.4. Surface Wettability of Silane-coated cellulose aerogel

**Figure 8:**
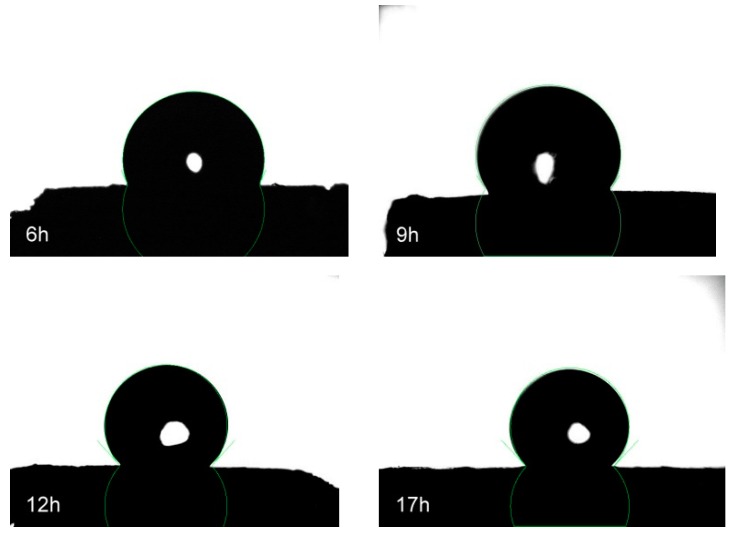
Measurements of water contact angle of cellulose aerogels after different coating processes.

Water contact angle measurements were also carried out on the uncoated and coated aerogel to study their surface wettability. The uncoated CA sample absorbed the distilled water droplet immediately in the test so no measurable contact angle was recorded. In contrast, high contact angles are observed for the MEMO silane-coated aerogel, as shown in Figure 8, proving the hydrophobicity of the material.

Table 2 shows the improvement of the water contact angle of cellulose aerogels from 114.1 ± 5.26° to 132.3 ± 4.68° when the silanization time was increased from 6 to 12 h, indicating the high hydrophobicity of the obtained SCA. No increase in water contact angle was observed with further extended reaction times.

### 3.5. Oil/Solvent Absorption Capacity of SCA and Its Recycleability

Owing to their low density, high porosity, and surface hydrophobicity, the silane-treated cellulose aerogels may be an ideal candidate for the selective absorption of oils and organic solvents from water. To examine the oil/solvent absorption behavior of MEMO silane-coated cellulose aerogel, several oils and organic solvents, such as toluene and gasoline were used. 

**Figure 9:**
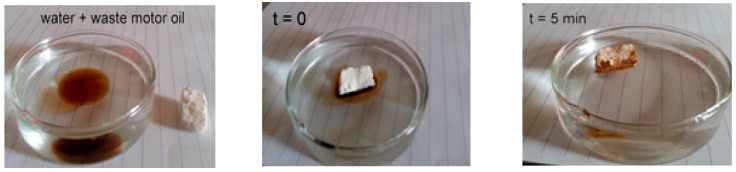
Waste motor oil absorption test of the silanized cellulose aerogel with MEMO silane.

**Figure 10:**
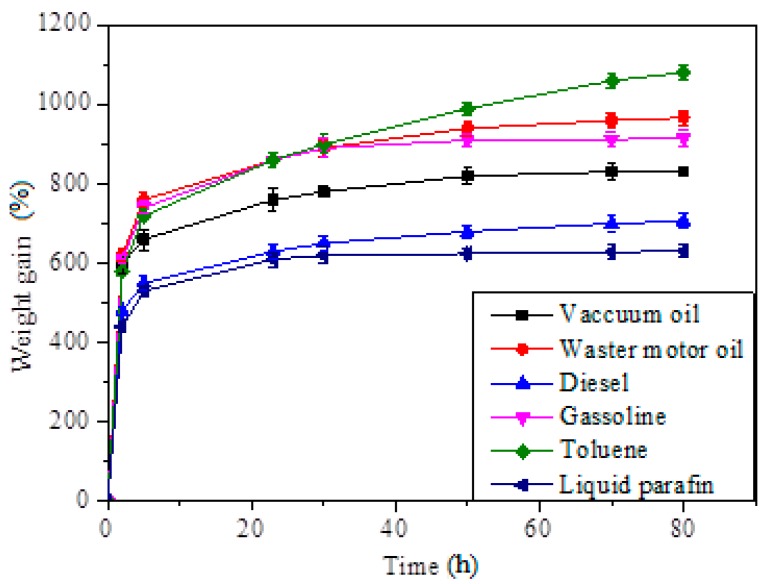
Absorption kinetics of oils and organic solvents on the silane-coated cellulose aerogel.

Figure 9 shows the first minutes of the waste motor oil sorption process. The material absorbed the motor oil easily while floating in water, indicating high capacity absorption of the aerogel. After 5 min, there was no trace of waste motor oil on the water, showing that the sorption was completed successfully. Figure 10 shows the sorption kinetics of the oils and solvents on the silane-coated cellulose aerogel. The absorption rates were quite high at the very first stage and saturation was achieved after 75 h for all types of oils and solvents.

**Figure 11:**
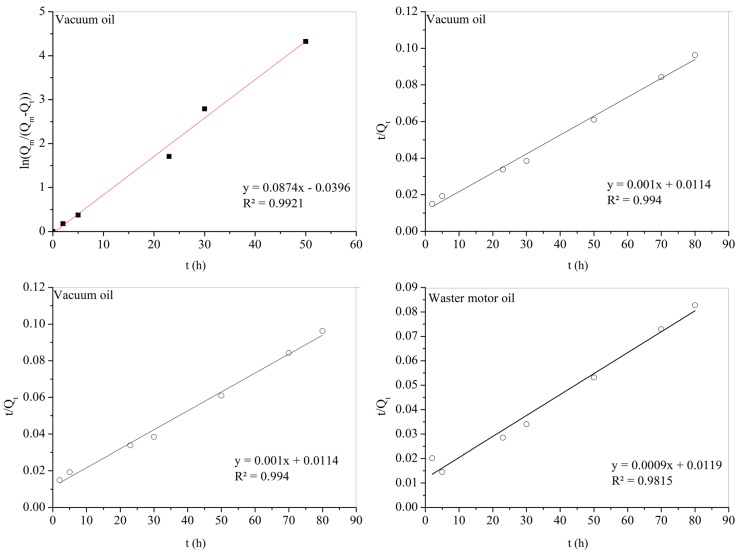
Pseudo-first-order and pseudo-second-order absorption linear fitting of the vacuum oil and waste motor oil onto SCA.

The experiment showed the linear relationship for both ln (*Q_m_*/(*Q_m_*−*Q_t_*)) and *t*/*Q_t_* versus absorption time, *t*, for two representative adsorbates: vacuum oil and waste motor oil as shown in Figure 11. These results mean that the adsorption kinetics of the SCA followed the pseudo-first-order and pseudo-first-order equation quite similarly. 

Equations (3) and (4) were used to calculate the absorption rate constants *k*_1_, *k*_2__,_ and correlation coefficient *R*^2^ from Figure 11 as seen in Table 3.

The results in Table 3 indicated that the *R*^2^ value of the pseudo second-order model of vacuum oil is higher than that of the pseudo first-order model. While the R^2^ value of the pseudo second-order model of waste motor oil is lower than that of the pseudo first-order model. These results mean that the pseudo second order model can predict better the oil absorption behavior for vacuum oil and the pseudo first order model is better for waste motor oil in this work. The absorption processing of vacuum oil is faster than the absorption of waster motor oil because the *k*_1_ and *k*_2_ values of vacuum oil is higher than those of waste motor oil.

**Figure 12:**
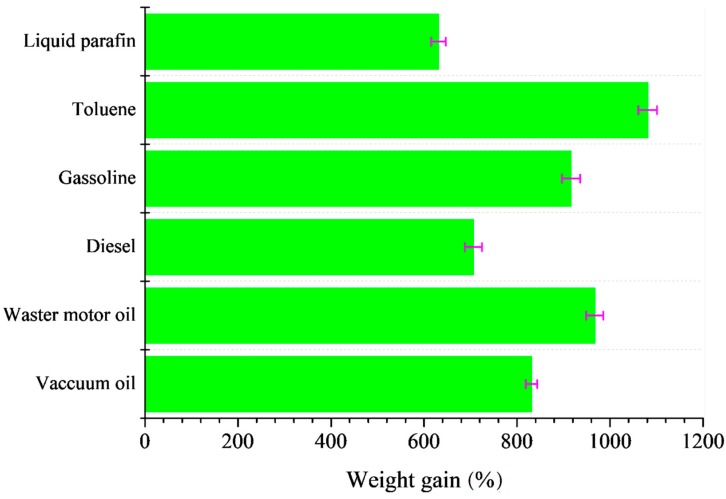
Absorption capacities of silane-coated cellulose aerogel for various oils and organic solvents as indicated by weight gain.

Figure 12 shows the maximum absorption capacities of the oils and organic solvents on the silane-coated cellulose aerogel. The results showed that the absorbent had sorption capacity ranging from 631 ± 15.9% to 1081 ± 20.1% by weight gain. The high oil/solvent absorption capability of the silane-coated cellulose aerogel can be attributed to its highly porous structure and hydrophobic silane coating. 

**Figure 13:**
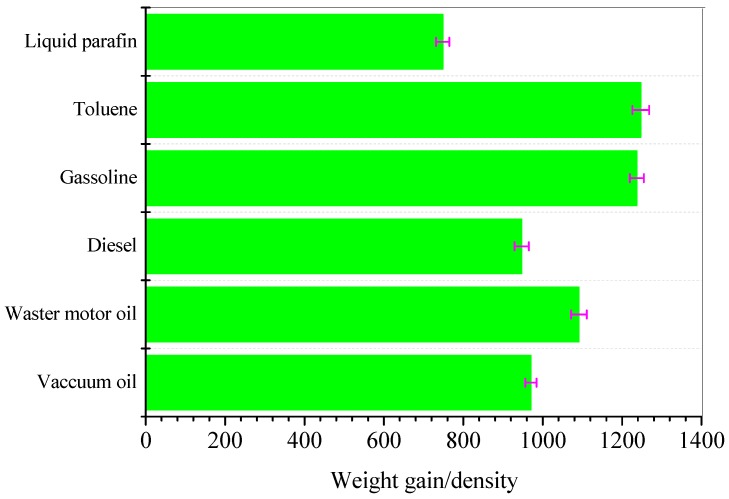
Absorption capacities normalized by the density of the respective oil or organic solvent.

Because the weight gain of aerogel is related to the density of the respective oils and organic solvents, it can be normalized by dividing the oils and organic weight gain by the density of each respective oil and organic solvent. The results are reported in Figure 13. As shown in Figure 13, the highest absorption capacity was found for toluene and gasoline probably because these organic solvents possess the lowest viscosity. A lower viscosity would facilitate the penetration of solvent into the porous network of the aerogel more easily, leading to a higher oil/solvent absorption capacity.

**Figure 14:**
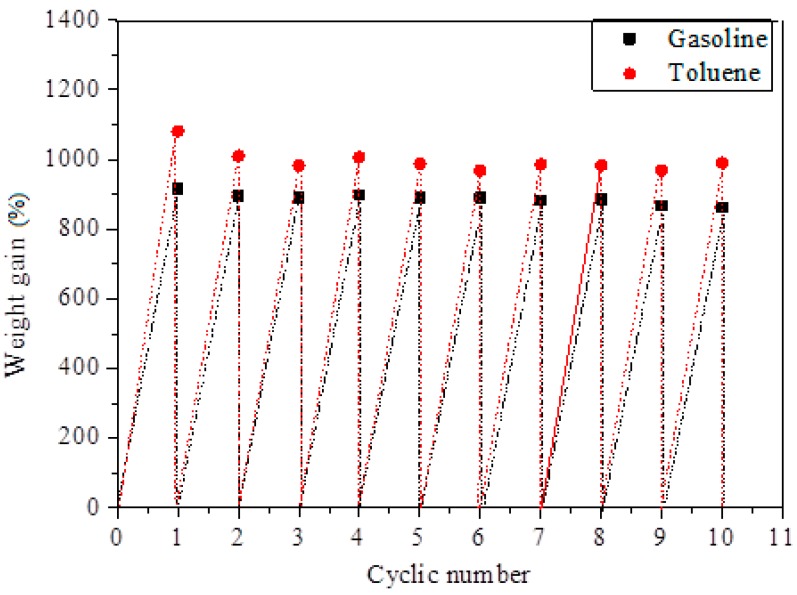
The cyclic adsorption capacity of sample SCA for gasoline and toluene.

Furthermore, for the recyclability test, the used absorbent was directly squeezed by hand and reused to absorb the oil and organic solvent. The absorption capacities of SCA for ten cycles are shown in Figure 14. 

After ten cycles, the adsorption capacity of the SCA for representative gasoline decreased from 916% to 862%. For toluene, the adsorption capacity decreased from 1081% to 989 % after ten cycles.

## 4. Conclusions

This paper reported the fabrication of a low density (0.084 g/cm^3^) and highly porous (94.5%) green aerogel for the cleaning of oil and organic solvents from micron-size white bamboo fibrils (MWBF) with a very simple alkaline/ urea mixture solution method followed by a common freeze-drying process. FT-IR and EDX characterization were used to examine the surface morphology and chemical compositions of the silane-modified cellulose aerogel. The coating with MEMO silane for oil absorption purposes made the cellulose aerogel highly hydrophobic with water contact angles larger than 132.3 ± 6.92° and exhibited high absorption capacities of 1091 ± 19.6%, 1237 ± 17.6%, and 1247 ± 21.1% by weight gain for waste motor oil, diesel, and gasoline, respectively. Based on these results, the modified aerogels can be used to clean up oil spills and toxic chemicals in aquatic environments with the recyclability over 10 times.

## Figures and Tables

**Table 1:** Physical properties of cellulose aerogels.

Sample	Cellulose Content (%)	Coagulation Solution	Density (g/cm^3^)	Porosity (%)	Surface Area (m^2^/g)
Cellulose aerogel	1.5	Ethanol	0.085	94.46	–
		Sulfuric acid	0.116	92.42	13.419
	2	Ethanol	0.131	91.41	–
		Sulfuric acid	0.135	91.19	9.046
	2.5	Ethanol	0.144	90.58	8.155

**Table 2:** Change of coated cellulose aerogel’s water contact angle with silianation time.

Silanization Time (h)	Contact Angle (°)
6	114.1 ± 5.26
9	125.5 ± 4.68
12	132.3 ± 6.92
17	131.8 ± 5.18

**Table 3:** Summary of the maximum oil absorption capacities and the absorption rate constants of the SCA using the pseudo-first-order and pseudo-second-order models.

Adsorbate	Maximum Absorption Capacity	Pseudo-First-Order		Pseudo-Second-Order	
	Q_m_ (%)	k_1_	R^2^	k_2_	R^2^
Vacuum oil	831 ± 12.2	0.0874	0.9921	1.27 × 10^−5^	0.994
Waste motor oil	968 ± 18.6	0.0707	0.9956	8.99 × 10^−5^	0.9815
